# Diagnostic accuracy of C-reactive protein and procalcitonin in suspected community-acquired pneumonia adults visiting emergency department and having a systematic thoracic CT scan

**DOI:** 10.1186/s13054-015-1083-6

**Published:** 2015-10-16

**Authors:** Josselin Le Bel, Pierre Hausfater, Camille Chenevier-Gobeaux, François-Xavier Blanc, Mikhael Benjoar, Cécile Ficko, Patrick Ray, Christophe Choquet, Xavier Duval, Yann-Erick Claessens, on behalf of the ESCAPED study group

**Affiliations:** Department of General Practice, University Paris Diderot, Sorbonne Paris Cité, 16 rue Henri Huchard, 75018 Paris, France; UMR 1137, INSERM, IAME, Paris, France; University Pierre et Marie Curie, Paris, France; Emergency Department, University Hospital Pitié-Salpêtrière, Assistance Publique-Hôpitaux de Paris (AP-HP), Paris, France; Department of Automated Biological Diagnosis, University Hospitals Cochin-Broca-Hôtel Dieu, HUPC, Assistance Publique-Hôpitaux de Paris (AP-HP), 75014 Paris, France; University of Nantes, Nantes, France; University Hospital Nantes, Institut du Thorax, Service de Pneumologie, Nantes, France; Department of Radiology, University Hospital Tenon, 75020 Paris, France; Infectious Disease Department, Bégin Military Teaching Hospital, 94163 Saint-Mandé cedex, France; Emergency Department, University Hospital Tenon, Assistance Publique-Hôpitaux de Paris (AP-HP), University Pierre et Marie Curie, 75020 Paris, France; Emergency Department, University Hospital Bichat-Claude Bernard, Assistance Publique-Hôpitaux de Paris (AP-HP), 75018 Paris, France; Inserm CIC 1425, University Hospital Bichat-Claude Bernard, Assistance Publique-Hôpitaux de Paris (AP-HP), 75018 Paris, France; University Paris Diderot, Sorbonne Paris Cité, 75018 Paris, France; Emergency Department, Hospital Princesse Grace, Monaco, Monaco

## Abstract

**Introduction:**

Community-acquired pneumonia (CAP) requires prompt treatment, but its diagnosis is complex. Improvement of bacterial CAP diagnosis by biomarkers has been evaluated using chest X-ray infiltrate as the CAP gold standard, producing conflicting results. We analyzed the diagnostic accuracy of biomarkers in suspected CAP adults visiting emergency departments for whom CAP diagnosis was established by an adjudication committee which founded its judgment on a systematic multidetector thoracic CT scan.

**Methods:**

In an ancillary study of a multi-center prospective study evaluating the impact of systematic thoracic CT scan on CAP diagnosis, sensitivity and specificity of C-reactive protein (CRP) and procalcitonin (PCT) were evaluated. Systematic nasopharyngeal multiplex respiratory virus PCR was performed at inclusion. An adjudication committee classified CAP diagnostic probability on a 4-level Likert scale, based on all available data.

**Results:**

Two hundred patients with suspected CAP were analyzed. The adjudication committee classified 98 patients (49.0 %) as definite CAP, 8 (4.0 %) as probable, 23 (11.5 %) as possible and excluded in 71 (35.5 %, including 29 patients with pulmonary infiltrates on chest X-ray). Among patients with radiological pulmonary infiltrate, 23 % were finally classified as excluded. Viruses were identified by PCR in 29 % of patients classified as definite. Area under the curve was 0.787 [95 % confidence interval (95 % CI), 0.717 to 0.857] for CRP and 0.655 (95 % CI, 0.570 to 0.739) for PCT to detect definite CAP. CRP threshold at 50 mg/L resulted in a positive predictive value of 0.76 and a negative predictive value of 0.75. No PCT cut-off resulted in satisfactory positive or negative predictive values. CRP and PCT accuracy was not improved by exclusion of the 25 (25.5 %) definite viral CAP cases.

**Conclusions:**

For patients with suspected CAP visiting emergency departments, diagnostic accuracy of CRP and PCT are insufficient to confirm the CAP diagnosis established using a gold standard that includes thoracic CT scan. Diagnostic accuracy of these biomarkers is also insufficient to distinguish bacterial CAP from viral CAP.

**Trial registration:**

ClinicalTrials.gov registry NCT01574066 (February 7, 2012)

**Electronic supplementary material:**

The online version of this article (doi:10.1186/s13054-015-1083-6) contains supplementary material, which is available to authorized users.

## Introduction

Community-acquired pneumonia (CAP) is a frequently seen disease, with high morbidity and mortality, accounting for 600,000 hospitalizations each year. It represents the seventh leading cause of death in the USA [1]. CAP prognosis depends on the rapidity of specific treatment, which should ideally be initiated within four hours and no later than eight hours after diagnosis [2, 3]. CAP diagnosis is based on the clustering of non-specific pulmonary and general symptoms [4, 5], an increase in biomarkers reflecting systemic inflammatory response syndrome (SIRS), and the presence of new parenchymal infiltrates on chest X-ray. However, CAP diagnosis remains uncertain in many cases with alternative diagnoses, such as cardiac failure, acute bronchitis, chronic obstructive pulmonary disease (COPD) exacerbations, pulmonary embolism, neoplasia, and sepsis [6, 7].

Part of the uncertainty of CAP diagnosis may be due to the high rate of chest X-ray misdiagnosis [8, 9]; over diagnosis of CAP is frequent when infiltrates of non-infectious origin coexist with pulmonary or general symptoms, and the diagnosis of CAP is often ignored when the lung infiltrates are at the limit of visibility or are hidden due to superposition [10]. We recently published a study in which thoracic CT scan was systematically performed in a population of clinically suspected CAP patients visiting the emergency department for CAP (the ESCAPED study) [11]. We showed that CAP diagnosis based on chest X-ray led to a false CAP diagnosis in many patients: among CAP suspected patients with radiological pulmonary infiltrate, CAP diagnosis was excluded in around 30 % of patients based on CT scan results; on the contrary, among patients without radiological pulmonary infiltrate, one-third had a pulmonary infiltrate on thoracic CT-scan. We also reported the isolation of viruses in one-third of patients [11, 12].

Several attempts have been made to improve CAP diagnosis based on biomarkers, such as C-reactive protein (CRP) and procalcitonin (PCT); however, there are conflicting data on their reliability [13–17]. This could be due to the consideration of CAP diagnosis based on chest X-ray as establishing pulmonary infection. In the present study, we aimed to analyze CRP and PCT values in the population of the ESCAPED study reported above for whom CAP diagnosis was established by an adjudication committee which founded its judgment on all usual available data, systematic multidetector thoracic CT scan performed at inclusion, and results from a day-28 follow-up. We also analyzed whether the viral etiology of definite CAP based on polymerase chain reaction (PCR) multiplex naso-pharyngeal swab interfered with the accuracy of the biomarkers.

## Methods

### Setting

ESCAPED was a multicenter, prospective, interventional study, entitled “Early Thoracic CT-Scan for Community-Acquired Pneumonia at the Emergency Department (ESCAPED)” [11], conducted from November 2011 to January 2013, in four emergency departments (EDs) of four tertiary teaching hospitals in Paris, France, designed to measure the impact of thoracic CT scan on clinical decision. The study was sponsored and monitored by the Paris public health hospitals, and funded by the French Ministry of Health. The French health authorities (Agence nationale de sécurité des medicaments et produits de santé, ANSM) and the institutional review board for the protection of human subjects approved the study protocol and patient informed consent procedures. All enrolled patients provided written informed consent for inclusion. The protocol was registered in the clinicaltrial.gov website under the PACSCAN acronym, the French translation of the English ESCAPED acronym (NCT01574066). The Ethics Committee of Ile de France (Comité de Protection des Personnes. Paris N° 2011-oct-12749) approved the study protocol.

### Objectives

The primary objective was to compare CRP and PCT values in the four different categories of CAP level of certainty using the day-28 adjudication committee classification. The four categories were: 1) absence of CAP hereafter referred to as excluded CAP diagnosis; 2) possible CAP; 3) probable CAP; and 4) definite CAP. The secondary objectives were to assess whether CRP and PCT were associated with CAP diagnosis using sensitivity analyses in three successive subgroups chosen a priori; 1) when specifically considering patients classified as having excluded CAP diagnosis and definite CAP (i.e., the patients for whom the level of certainty was the highest); 2) when patients with excluded CAP diagnosis and diagnosed extra-pulmonary infectious disease (which may increase biomarker values) were not taken into account, in the excluded CAP group; and 3) when patients classified as viral CAP were not taken into account in the definite CAP group, as PCT has been reported to be lower in viral infections as compared to bacterial infections [18].

### Study population

Consecutive adults (18 years of age and above) visiting the participating EDs were enrolled if the attending emergency physician clinically suspected CAP. Clinical suspicion of CAP was based on the investigator’s own judgment and had to fulfill the following criteria: new onset of systemic features (at least one among: sweat, chills, aches and pain, temperature ≥38 °C or <36 °C) and symptoms of an acute lower respiratory tract illness (at least one among: cough, sputum production, dyspnea, chest pain, altered breathing sounds at auscultation). Pregnant women, patients in palliative care or with anticipated barriers to completing follow-up data collection, patients classified ≥3 according to the CRB65 score and those requiring intensive care for any purpose, due to specific management of critically ill CAP patients, were not eligible. This study examined patients from the ESCAPED study, for whom the CRP and PCT values and the multiplex PCR results were all available.

### Patient management and data collection

Patient management was based on local practices in the emergency departments. No recommendation was given concerning the performance of CRP and PCT dosage, as no dosages are recommended in French CAP guidelines. Recorded baseline data consisted of demographic data (age, gender), coexisting illnesses, symptoms, clinical findings and laboratory tests. For each individual, CRB65 and Pneumonia Severity Index (PSI) were calculated [19].

### Radiological data and CAP diagnosis classification

Multidetector thoracic CT-scan was performed after chest X-ray, ideally within the four hours following inclusion. Chest X-ray and thoracic CT-scan were performed using a standardized protocol. The four levels of CAP probability according to CT scan were defined as definite (systematic alveolar condensation, alveolar condensation with peripheral and localized ground glass opacities, bronchiolar focal or multifocal micronodules), probable (peripheral alveolar condensation, retractile systematic alveolar condensation, or diffuse ground glass opacities), possible (pulmonary infarct), or excluded (pulmonary mass, other abnormalities, or normal images). Scan views were recorded on a DVD.

### Adjudication committee

Based on data collected from baseline standardized case report forms, DVD recorded pictures of X-ray and CT-scan, and blinded to local interpretations, an adjudication committee consisting of three independent senior experts in infectious diseases, pneumology and radiology retrospectively assigned the probability of CAP diagnosis using the same 4-level Likert scale, with all available data including patients’ discharge summary, and follow-up data obtained by assistant investigators who contacted by phone either the patient, relatives or general practitioners at day 28. For this study, the gold standard of CAP was the diagnosis assessed by this adjudication committee. Alternative diagnoses were established for excluded CAP and classified as non-CAP pulmonary diseases and extra-pulmonary infectious diseases and others.

### Biomarker measurements

Blood samples were collected at inclusion in sodium heparin-treated tubes, centrifuged, and stored at −40 °C until completion of the study. CRP and PCT concentrations were measured *a posteriori* on plasma collection (see Additional file 1 for methodology), except for patients in whom marker dosage was performed by the emergency practitioner on his own initiative.

### Microbiological samples and microbial CAP classification

Naso-pharyngeal swabs were collected at enrollment and placed in a Middle Virocult MWE (Sigma®) transport medium. Samples were kept at room temperature and sent to the virology laboratory of Bichat - Claude Bernard Hospital (Paris) as soon as possible after collection. The samples were not frozen and thawed. Multiplex PCR (RespiFinder-19 assay (Pathofinder®, Maastricht, Netherlands)) was performed on naso-pharyngeal swabs to detect 15 respiratory viruses - *coronavirus 229E*, *NL63*, *OC43*, *human metapneumovirus (hMPV)*, *influenza A*, *A (H1N1) pdm2009* and *B viruses*, *parainfluenza viruses 1*, *2*, *3*, and *4*, *respiratory syncytial virus (RSV) A and B*, *rhinovirus*, *adenovirus*, and 4 intracellular bacteria - *Bordetella pertussis*, *Chlamydophila pneumoniae*, *Legionella pneumophila*, *Mycoplasma pneumoniae*, in one reaction. The multiplex PCR results were not available to the adjudication committee. Routine microbiological examinations were also performed at the discretion of the emergency physicians and included blood culture, sputum culture, and antigenuria (see Additional file 1 for methodology).

CAP, classified as definite, was considered as being of viral origin when multiplex PCR was positive for at least one of the 15 respiratory viruses and no bacteria were found using PCR and routine bacterial microbiological samples (sputum, blood culture, antigenuria) when performed.

### Statistical analysis

Baseline and follow-up characteristics were described by means and standard deviations (SD) or by median and interquartile range (IQR) for continuous variables normally distributed or with skewed distribution, respectively, and by percentages for categorical variables, for the total study population and for the study groups. We performed chi-square or Fisher exact tests when appropriate for qualitative variables, and the Student or Mann–Whitney tests for continuous variables with skewed distributions to compare baseline patient characteristics and study outcomes between study groups.

The distribution values of the biomarkers were determined in the different populations of patients using boxplots. The performances of CRP and PCT in predicting definite CAP were evaluated by sensitivity analysis (definite CAP vs excluded CAP). CRP was evaluated at several cut-off points of 20 mg/L, 30 mg/L, 50 mg/L, 70 mg/L, and 100 mg/L, values used in previous studies [15, 20, 21]. Several cut-off points for PCT were chosen at the level of 0.10 μg/L [18], and at the two levels for suspected bacterial infection as stated by the manufacturer, i.e., 0.25 μg/L and 0.50 μg/L. Sensitivities, specificities, positive predictive values (PPVs), negative predictive values (NPVs), and likelihood ratio were calculated. Receiver operating characteristic (ROC) curves were drawn, area under the curve AUC was computed and optimal cut-off was identified by the maximization of the Youden’s index, comparing biomarker values in patients with excluded CAP and definite CAP. From these optimal cut-offs for CRP and PCT, sensitivity analyses were performed combining the CRP and PCT cut-offs.

A multivariate logistic regression model was built to identify factors associated with having definite CAP as compared to having an excluded CAP diagnosis. We excluded from the excluded CAP diagnosis group, patients with an extra-pulmonary infectious disease. All variables with a p value of < 0.25 in the bivariate analysis were entered into a multivariate logistic regression with a backward stepwise approach; the discrimination was evaluated by the C-index and its 95 % confidence interval (95 % CI) and the calibration was evaluated by the Hosmer Lemeshow goodness-of-fit test.

All tests were two-sided, and p-values below 0.05 were considered to denote statistical significance. All statistical analyses were performed using SPSS statistical software version 21.0 (SPSS Inc., Chicago, IL, USA).

## Results

**Fig. 1:**
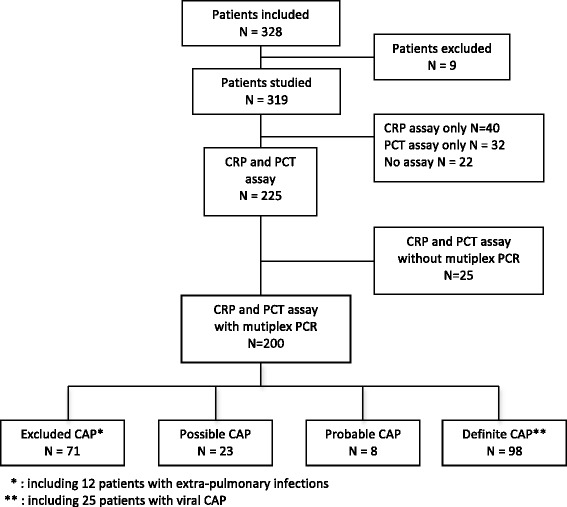
Flow chart of the studied population according to the day 28 adjudication committee classification *CAP* community-acquired pneumonia, *CRP* C-reactive protein, *PCT* procalcitonin

Two hundred patients with suspected CAP out of the 319 in the ESCAPED study were included in the present study, for which CRP and PCT assays and nasopharyngeal swab for multiplex PCR were available (Fig. 1). Characteristics of the 200 patients (age, age more than 65, gender, probability of CAP diagnosis by adjudication committee) were not significantly different from those of the 119 other patients of the ESCAPED study and are summarized in Table 1. CRP and PCT assays were performed based on the emergency practitioner’s own initiative in 70 patients for CRP and 131 for PCT, or performed a posteriori on plasma samples of the remaining patients. Sex ratio was approximately 1. More than half of the patients (54 %) were 65 years of age or older. The number of patients suffering from significant underlying disorders was 102 (51 %), including 57 (28 %) with pulmonary disorders. Cough (n = 153, 76 %) and dyspnea (n = 142, 71 %) were the most frequent symptoms. Pulmonary auscultation detected unilateral crackles in 65 (32 %), and 96 (48 %) patients had expectoration.

**Table 1:** Characteristics of the 200 patients of the study

	No (%) or mean ± SD^a^
Characteristics	Total (n = 200)
General characteristics	
Age	
Mean age (years)	63.9 ± 19.1
≥ 65 years	108 (54.0)
Sex	
Female	99 (49.5)
Male	101 (50.6)
Nursing home resident	8 (4.0)
Background and vaccinations	
Comorbidities	
At least 1 comorbidity	102 (51.0)
Chronic respiratory disease	57 (28.5)
Congestive heart failure	16 (8.0)
Kidney disease	13 (6.5)
Neoplasia	18 (9.0)
Liver disease	9 (4.5)
History of stroke	7 (3.5)
Vaccination status	
Influenza vaccination during the past year	75 (37.5)
Pneumococcal vaccination	27 (13.5)
Community-acquired pneumonia characteristics at inclusion	
Previous antibiotic treatment	68 (34.0)
Symptoms duration before visiting ED (days)	9.6 ± 10.9
Signs and symptoms in the ED	
Cough	153 (76.5)
Chest pain	66 (33.0)
Expectoration	96 (48.0)
Dyspnea	142 (71.0)
Chills	71 (35.5)
Headaches	43 (21.5)
Myalgia	45 (22.5)
Crackles	65 (32.5)
Fever	63 (31.5)
Confusion	3 (1.5)
Respiratory rate > 30/min	24 (12.0)
Heart rate > 125/min	13 (6.5)
Systolic blood pressure < 90 mmHg	4 (2.0)
Diastolic blood pressure < 60 mmHg	16 (8.0)
Community-acquired pneumonia severity scores	
PSI risk class	
I	31 (15.5)
II	61 (30.5)
III	41 (20.5)
IV	54 (27.0)
V	13 (6.5)
Biological data	
White blood cell (10^3^/mm^3^)	11.6 ± 5.0
Urea > 11 mmol/L	23 (11.5)
pH < 7.35	2 (1.0)
PaO_2_ < 60 mmHg or Sat0_2_ < 90 %	25 (12.5)
Biomarkers results^a^	
CRP (mg/L)	
In all patients (n = 200)	74.5 [21.6 - 150.8]
In patients with a CAP classified as	
« excluded » (n = 71)	23.4 [5.0 - 96.2]
« possible » (n = 23)	48.6 [16.0 - 147.1]
« probable » (n = 8)	78.8 [27.7 - 240.9]
« definite » (n = 98)	125.1 [65.0 - 208.7]
Procalcitonin (PCT) (μg/L)	
In all patients (n = 200)	0.18 [0.07 - 0.91]
In patients with a CAP classified	
« excluded » (n = 71)	0.11 [0.06 - 0.42]
« possible » (n = 23)	0.14 [0.07 - 0.63]
« probable » (n = 8)	0.63 [0.06 - 1.41]
« definite » (n = 98)	0.24 [0.11 - 1.38]
Community-acquired pneumonia management	
Emergency physician's mean years in practice	5.8 ± 6.0
28-day mortality	6 (3.0)

Abbreviations: *ED* emergency department, PSI Pneumonia Severity Index, *CRP* C-reactive protein, *CAP* community-acquired pneumonia

^a^Results are expressed as number (%) or mean ± standard deviation (SD) except for biomarker results expressed as median (IQR)

### Chest X-ray results and CT scan results

Pulmonary infiltrates were seen on chest X-ray in 127 (63.5 %) patients. Thoracic CT-scan excluded a CAP diagnosis in 16.5 % of these 127 patients; on the contrary, thoracic CT-scan revealed a parenchymal infiltrate in 27 % of the 73 patients without infiltrate on chest X-ray.

### Day-28 adjudication committee classification

Based on all available data including multidetector CT scan results (but excluding PCR results), the adjudication committee classified CAP as excluded in 71 (35.5 %), possible in 23 (11.5 %), probable in 8 (4.0 %), and definite in 98 patients (49 %). Among the 71 excluded CAP diagnoses, 59 were categorized as non-CAP pulmonary diseases (neoplasia, acute bronchitis, emphysema, COPD, pulmonary embolism, acute pulmonary edema, tuberculosis, miscellaneous) and 12 as extra-pulmonary infectious diseases (urinary tract infections, septicemia, discitis, meningitis, erysipela, acute sinusitis infection and peritonitis). Bacterial and viral data of patients with a definite CAP classification are presented in Additional file 2.

### Biomarker results

**Fig. 2:**
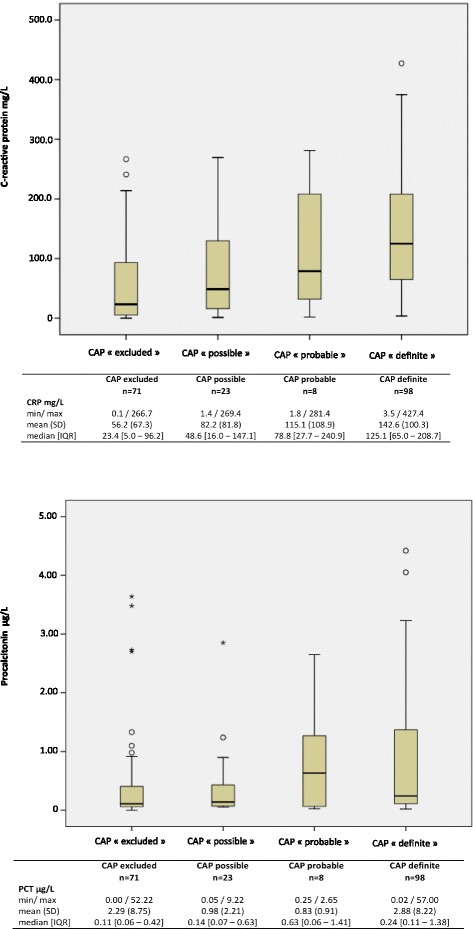
C-reactive protein (CRP) (*upper panel*) and procalcitonin (PCT) (*lower panel*) boxplot for patients according to each level of community-acquired pneumonia diagnosis certainty classification. PCT values greater than 5 μg/L are not shown

The CRP and PCT distributions in the 200 patients are presented in Fig. 2 according to the adjudication committee CAP classification. The median CRP value increased progressively from 23.4 mg/L [5.0 – 96.2 (excluded CAP)] to 125.1 mg/L [65.0–208.7 (definite CAP)] (p < 0.01), as did median PCT values [from 0.11 μg/L (0.06 – 0.42) to 0.24 μg/L (0.11 – 1.38), respectively; p < 0.01].

**Fig. 3:**
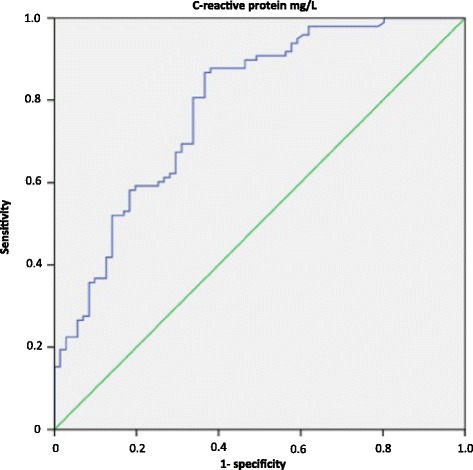
C-reactive protein ROC curves predicting definite community-acquired pneumonia diagnosis. AUC = 0.787. 95 % CI = 0.717 to 0.857. Youden’s index = 0.501 for an optimal CRP cut-off point at 45.9 mg/L *ROC* receiver operating characteristic, *AUC* area under the curve, *CI* confidence interval, *CRP* C-reactive protein

**Fig. 4:**
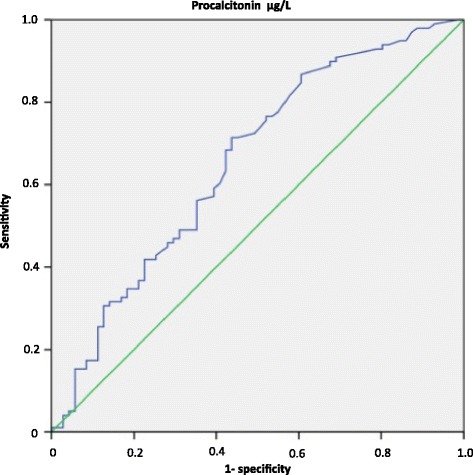
Procalcitonin ROC curve predicting definite community-acquired pneumonia diagnosis. AUC = 0.655. 95 % CI = 0.570 to 0.739. Youden’s index = 0.307 for an optimal PCT cut-off point at 0.13 μg/L *ROC* receiver operative characteristic, *AUC* area under the curve, *CI* confidence interval, *PCT* procalcitonin

A statistically significant difference between the two groups (excluded CAP vs definite CAP) was demonstrated for several cut-off points for CRP and PCT (Table 2). For CRP, the value of 50 mg/L resulted in a PPV of 0.76 and a NPV of 0.75. For PCT, no value resulted in a satisfactory PPV or NPV. For these two biochemical markers, the ability to predict CAP was evaluated by a ROC curve. The AUC was 0.787 (95 % CI 0.717-0.857), optimal cut-off = 45.9 mg/L for CRP (Fig. 3) and 0.655 (95 % CI 0.570-0.739), optimal cut-off = 0.13 μg/L for PCT (Fig. 4).

**Table 2:** Sensitivity, specificity, PPV and NPV according to different C-reactive protein (CRP) and procalcitonin (PCT) cut offs in the patients with excluded or definite community acquired pneumonia

Biomarkers' cut-off	Total	Excluded CAP	Definite CAP	p value	Se	Sp	PPV	NPV	LR+	AUC
	(N = 169)	(N = 71)	(N = 98)							
	n (%)	n (%)	n (%)							
CRP cut-off										
> 20 mg/L	133 (79)	41 (58)	92 (94)	<0.001	93.9	42.2	69.2	83.3	1.62	
> 30 mg/L	123 (73)	35 (49)	88 (90)	<0.001	89.8	50.7	71.5	78.2	1.82	
> 50 mg/L	109 (64)	26 (37)	83 (85)	<0.001	84.7	63.4	76.1	75.0	2 .31	0.787
> 70 mg/L	92 (54)	24 (34)	68 (69)	<0.001	69.4	66.2	73.9	61.0	2.05	
> 100 mg/L	73 (43)	15 (21)	58 (59)	<0.001	59.2	78.9	79.4	58.3	2.80	
PCT cut-off										
> 0.10 μg/L	115 (68)	39 (55)	76 (78)	0.003	77.5	45.1	66.1	59.2	1.41	
> 0.25 μg/L	74 (44)	25 (35)	49 (50)	0.061	50.0	64.7	66.2	48.4	1.41	0.655
> 0.50 μg/L	53 (31)	16 (23)	37 (38)	0.044	37.7	77.5	69.8	47.4	1.67	
CRP >49.5 mg/L and PCT cut-off combined										
PCT > 0.13 μg/L	83 (49)	21 (29)	62 (63)	<0.001	63.2	70.4	74.6	58.1	2.13	
PCT > 0.1 μg/L	90 (53)	22 (31)	68 (69)	<0.001	69.4	69.0	75.5	62.0	2.23	
PCT > 0.25 μg/L	68 (40)	21 (29)	47 (48)	0.018	47.9	70.4	69.1	49.5	1.62	
PCT > 0.5 μg/L	51 (30)	15 (21)	36 (36)	0.041	36.7	78.9	70.6	47.4	1.74	

Abbreviations: *CAP* community acquired pneumonia, *Se* sensitivity, *Sp* specificity, *PPV* positive predictive value, *NPV* negative predictive value, *LR* likelihood ratio, *AUC* area under the curve

Sensitivity analyses for the combination of CRP and PCT, using these optimal cut-offs, resulted in a PPV of 0.74 and a NPV of 0.58. Use of the other PCT cut-offs did not result in better PPV or NPV (Table 2).

### Impact of exclusion of patients with extra-pulmonary infections from the excluded CAP group

The exclusion of the 12 patients with extra-pulmonary bacterial infections from the 71 excluded CAP patients led to a non statistically significant decrease of the median CRP values [17.3 mg/L (3.6-57.5) (*p* = 0.203)] and PCT values [0.09 μg/L (0.06-0.27) (*p* = 0.309)] of the 59 remaining excluded CAP patients (see Additional file 3); the AUC also increased to 0.851 (95 % CI 0.790-0.913) for CRP and to 0.718 (95 % CI 0.636-0.799) for PCT, without bettering predictive performances for CAP (see Additional file 4). In the multivariate analysis, the presence of fever [OR 3.15 (1.29-7.73), *p* = 0.012] and the increase in CRP level [odds ratio (OR) 1.02 (1.01-1.03), *p* <0.001, for each mg] were independently associated with definite CAP whereas PCT increase was not (Table 3). The C-index of the final model was 0.862 (95 % CI 0.802-0.921) and the Hosmer Lemeshow p value was 0.002.

**Table 3:** Univariate and multivariate analysis of the clinical characteristics of excluded CAP patients without extra-pulmonary infections compared to definite CAP patients

Patient characteristics n (%) or median IQR	Total	Excluded CAP^a^	Definite CAP	*p*	OR	*p*
	N = 157	N = 59	N = 98		[95 % CI]	
Cough	122 (77.7)	41 (69.5)	81 (82.7)	**0.047**	-	
Chest pain	53 (33.7)	16 (27.1)	37 (37.8)	**0.168**	-	
Expectoration	78 (49.7)	27 (45.8)	51 (52.0)	0.509	-	
Dyspnea	111 (70.7)	44 (74.6)	67 (68.4)	0.471	-	
Chills	52 (33.1)	21 (35.6)	31 (31.6)	0.727	-	
Headaches	33 (21.0)	9 (15.2)	24 (24.5)	**0.225**	-	
Myalgia	35 (22.3)	11 (18.6)	24 (24.5)	0.432	-	
Crackles	49 (31.2)	13 (22.0)	36 (36.7)	**0.051**	-	
Fever	51 (32.5)	10 (16.9)	41 (41.8))	**0.001**	3.15 [1.29-7.73]	0.012
Confusion	0	0	0	-	-	
Respiratory rate > 30/min	19 (12.1)	7 (11.9)	12 ()	0.856	-	
Heart rate > 125/min	11 (7.0)	2 (3.4)	9 (9.2)	**0.211**	-	
Systolic blood pressure < 90 mmHg	3 (1.9)	0	3 (3.1)	0.292	-	
Diastolic blood pressure < 60 mmHg	10 (6.4)	1 ()	9 (9.2)	**0.091**	-	
White blood cells > 10.10^3^ /mm^3^	86 (54.8)	22 (37.3)	64 (65.3)	**0.004**	-	
PaO_2_ < 60 mmHg or Sat0_2_ < 90 %	18 (11.4)	6 (10.1)	12 (12.2)	0.799	-	
CRP	74 [21.3 – 146.1]	17.3 [3.6 – 57.5]	125.1 [65.0 - 208.7]	**<0.001**	1.02 [1.01-1.03]	<0.001
PCT	0.17 [0.07 – 0.72]	0.09 [0.06 - 0.28]	0.24 [0.11 - 1.38]	**<0.001**	-	-

*CAP* community-acquired pneumonia, *CRP* C-reactive protein, *PCT* procalcitonin

^a^Patients with excluded CAP without extra-pulmonary infections

The bold data correspond to the variables included into the multivariate logistic regression

### Impact of multiplex PCR results on biomarkers’ accuracy

Naso-pharyngeal multiplex PCR detected a microorganism in 61 of the 200 patients (30.5 %), including 33 out of the 98 definite CAP (see Additional file 2). Among these latter 33 patients, intracellular bacteria were identified in 4 (Mycoplasma pneumoniae in all), respiratory virus and bacteria in 4 (*Streptococcus pneumoniae + Influenza A virus (1), Streptococcus pneumoniae + Rhinovirus (1), Enterobacterieaceae + Rhinovirus (1), Intracellular bacteria + Influenza A virus (1)*), and respiratory virus alone in 25 (*Influenza A virus (8), Influenza B virus (2), Parainfluenza virus (3), Coronavirus (2), Rhinovirus (2), respiratory syncytial virus A (2), respiratory syncytial virus B (2), Metapneumovirus (2), Adenovirus (1), Coronavirus + Metapneumovirus (1))*. For the 25 patients with viral CAP, the median CRP value was 124.7 mg/L (68.9-223.0), and median PCT value was 0.46 μg/L (0.18-1.97). In definite CAP patients, the exclusion of these 25 patients with viral CAP led to a non statistically significant increase of the median CRP value 125.5 mg/L (63.3-209.5) (*p* = 0.93) but an unexpected decrease of the median PCT value 0.21 μg/L (0.09-1.33) (*p* = 0.49) of the 73 remaining definite CAP patients, and an AUC decrease to 0.847 (95 % CI 0.781-0.913) for CRP and to 0.687 (95 % CI 0.597-0.777) for PCT.

## Discussion

The present study is novel as patients prospectively benefited from extensive investigation to determine the diagnosis of CAP in the ED, including both early multidetector thoracic CT-scan and day-28 adjudication committee. This led to the correction of CAP diagnosis previously based on chest X-ray in a high number of patients. In these extensively characterized patients, both CRP and PCT lacked operational precision to allow the decision-making process to rule out or confirm diagnosis of CAP even in selected subgroups.

The clinical characteristics of the patients included in this sub-study are consistent with those in the current literature. As previously reported, patients frequently had a history of respiratory disorders, cancer and congestive heart failure [21, 22]. The design of the ESCAPED study required exclusion of patients within the highest CRB 65 categories, which limited the inclusion of patients older than 65. This may explain why the mean age of our patients (64 years) falls within the lower values of those reported elsewhere [19]. Data to identify the microbial agent responsible for the disease were collected by the usual techniques and multiplex PCR. Viral identification using naso-pharyngeal PCR that revealed viral respiratory infection in approximately one-third of cases was concordant with values reported in the literature [23]. Therefore, we believe that our results can be extrapolated to most emergency patients suffering from CAP.

In the present study, patients were recruited on the basis of initial clinical assessment for the diagnosis of CAP. Therefore, we believe that the characteristics of the patients closely correspond to those that lead practitioners to consider a possible diagnosis of CAP. In these patients, the design of our study allowed us to confirm or refute CAP diagnosis with a high level of certainty. Results confirmed the poor predictive value of clinical symptoms (new onset of systemic features and symptoms of an acute lower respiratory tract illness) in identifying CAP patients [21]. Indeed, clinical presentation of excluded CAP patients was similar to that of definite CAP patients except for fever and cough that were more frequent in definite CAP patients. Furthermore, the design also revealed that the combination of clinical symptoms and chest X-ray results led to CAP misdiagnosis in a high number of patients, including the 98 whose CAP diagnosis was excluded by the adjudication committee and who would have been considered as possible, probable or definite CAP without the use of the CT scan. This low specificity of clinical-standard radiological evaluation led to the consideration of either non-infectious pulmonary diseases (such as, cardiac failure, pulmonary embolism, pulmonary neoplasia or bronchitis) or extra-pulmonary infectious diseases as CAP. Of note, some of these diseases are also associated with increased biomarker values. This raises concerns about previous evaluations of biomarkers in CAP-suspected patients, which used clinical and standard radiological (chest X-ray) evaluations as the gold standard for CAP diagnosis [15].

The use of biomarkers has been advocated to improve diagnosis and management of patients with lower respiratory tract infections [14]. However, this issue is still unresolved [24], with conflicting positions [14, 15, 25, 26]. In our study, while median values of both biomarkers did increase with level of certainty for CAP diagnosis, we were unable to establish discriminating values for PCT. Recent data suggested that CRP could be of more help in assisting in the diagnosis of lower respiratory tract infections (LRTI) [15, 27, 28]. In our study, although CRP seems more discriminating than PCT, neither the experimental exclusion of extra-pulmonary bacterial infections from the excluded CAP group, nor the exclusion of viral CAP from the definite CAP patients group, made possible the determination of a discriminant cutoff. The combination of CRP and PCT was not more discriminating than each biomarker separately. An operational algorithm has been released to assist physicians in prescribing antimicrobial therapy [14, 26, 29]. According to this strategy, a PCT concentration higher than 0.25 μg/L should prompt administration of antibiotics to patients with suspected LRTI. In our study, this value was associated with poor performance. Additionally, mean PCT levels remained above this threshold both in excluded CAP patients without infectious disorders and in definite CAP presumably related to virus. Therefore, the gold standard for the diagnosis of CAP may influence the performance and utility of PCT in this setting.

This study has some limitations. First, the adjudication committee was not blinded to the value of biomarkers measured at bedside in some patients (70 for CRP and 131 for PCT) and its CAP classification could thus have been influenced by these results. However, the lack of statistically significant differences in the mean CRP and PCT values in the definite CAP cases, whether or not these biomarkers were available for the adjudication committee, argues against a major impact of these results on adjudication committee classification. Second, another critical point is the prescription of antibiotic therapy (34 %) previous to inclusion. We cannot exclude that these previously-treated CAP patients may have altered biomarker performance and reduced the yield of bacterial cultures, although such a population reflects the usual emergency department practice. Third, multiplex PCR was performed on naso-pharyngeal sampling and not on lower respiratory tract samples, which does not allow definite confirmation of the viral origin of CAP. However, a recent large study on CAP patients which reported a viral etiology of CAP at a comparable rate, did not find upper respiratory tract shedding in a control population without CAP explored during the same year and season [30]. Finally, even if multidetector thoracic CT scan is a better imaging examination than X-ray to explore the chest, only invasive local microbiological samples would have provided a diagnosis with certainty.

## Conclusions

Given the diversity of the clinical and radiological CAP presentations, CAP diagnosis is often uncertain. In our population of patients treated in the emergency room with clinical symptoms evoking CAP, neither CRP nor PCT cut-off values carried sufficient weight to confirm or refute CAP diagnosis at bedside; this underlines that these biomarkers are telltales of the host inflammatory response to the intrusion of microorganisms independent of the site of infection. These results, based on a systematic thoracic CT scan evaluation of CAP-suspected patients, do not argue for the use of CRP and PCT in routine care to diagnose CAP with certainty in patients visiting the ED for suspected CAP.

## Key messages

- The predictive value of clinical symptoms in identifying CAP patients is poor
- No CRP or PCT cut-off value is sufficiently discriminating to confirm or refute CAP diagnosis with a high level of certainty
- The diagnostic accuracy of biomarkers was not improved when CAP cases considered as viral were excluded from analysis.

## Additional files

Additional file 1:
**Descriptions of biomarker analysis methods and Multiplex PCR methods.** (DOC 24 kb) Additional file 2:
**Bacterial and viral data for patients with definite CAP.** (DOC 38 kb) Additional file 3:
**C-reactive protein and procalcitonin boxplot for patients with excluded CAP according to each category of alternative diagnosis.** (PDF 174 kb) Additional file 4:
**C-reactive protein and procalcitonin ROC curves predicting definite community-acquired pneumonia diagnosis (definite community acquired pneumonia versus excluded community acquired pneumonia without extra-pulmonary infections).** (PDF 176 kb)

## Abbreviations

CAP: Community-acquired pneumonia
COPD: Chronic obstructive pulmonary disease
CRP: C-reactive protein
IQR: Interquartile range
LRTI: Lower respiratory tract infections
NPV: Negative predictive value
OR: Odds ratio
PCR: Polymerase chain reaction
PCT: Procalcitonin
PPV: Positive predictive value
SD: Standard deviation
SIRS: Systemic inflammatory response syndrome
