# Supplementary material for: Diagnostic accuracy of C-reactive protein and procalcitonin in suspected community-acquired pneumonia adults visiting emergency department and having a systematic thoracic CT scan
Source: Crit Care. 2015 Oct 16;19:366. doi: 10.1186/s13054-015-1083-6 (PMC4608327; doi:10.1186/s13054-015-1083-6)
Supplement: Additional file 1: — Descriptions of biomarker analysis methods and Multiplex PCR methods. (DOC 24 kb) [file 13054_2015_1083_MOESM1_ESM.doc]

**Additional file 1**

**Biomarkers analysis methods:**

CRP concentrations were measured using the Tina-quant CRP-Gen3 immunoturbidimetric assay, on a Modular PP analyzer (Roche Diagnostics Meylan, France). PCT concentrations were analyzed using an electrochemiluminescent immunoassay (ELECSYS BRAHMS procalcitonin, Hennigsdorf, Germany) performed on a Cobas e601 analyzer (Roche Diagnostics, Meylan, France). For CRP, the measuring range was 0.6-350 mg/L. Coefficients of variation (obtained from the 2 quality controls) for CRP were <3% during the study period. The upper reference limit (URL) announced by the manufacturer was <5 mg/L. For PCT, the measuring range was 0.02-80 µg/L. Coefficients of variation (obtained from the 2 quality controls) for PCT were <4% during the study period. The upper reference limit (URL) announced by the manufacturer was 0.046 µg/L.

**Multiplex PCR methods:**

The assay involves a pre-amplification step, which combines reverse transcriptase and multiplex target amplification PCR, followed by a probe hybridization, ligation and amplification as described by the manufacturer. The RespiFinder-19 analyzes the amplified PCR products by capillary electrophoresis using a DNA analyzer (ABI 3130, Applied Biosystems®, Darmststadt, Germany) and provides diagnosis within 6 hours. The multiplex PCR method used in the present study was validated by the virology lab that participates every year in the European quality control program (QCMD) for each respiratory virus.
