# Supplementary material for: Diagnostic accuracy of C-reactive protein and procalcitonin in suspected community-acquired pneumonia adults visiting emergency department and having a systematic thoracic CT scan
Source: Crit Care. 2015 Oct 16;19:366. doi: 10.1186/s13054-015-1083-6 (PMC4608327; doi:10.1186/s13054-015-1083-6)
Supplement: Additional file 2: — Bacterial and viral data for patients with definite CAP. (DOC 38 kb) [file 13054_2015_1083_MOESM2_ESM.doc]

Additional file 2: viral and bacterial data for patients classified as “definite CAP”

| Viral and bacterial data | Patients classified as definite CAP  N=98 |
| --- | --- |
| **Virus identified by multiplex PCR** |  |
| Influenza A virus | 10^a,b^ |
| Influenza B virus | 2 |
| Parainfluenza virus | 3 |
| Coronavirus | 3^c^ |
| Rhinovirus | 4^d,e^ |
| Respiratory syncytial virus A | 2 |
| Respiratory syncytial virus B | 2 |
| Metapneumovirus | 3^c^ |
| Adenovirus | 1 |
| **Bacteria identified by multiplex PCR** |  |
| Mycoplasma pneumoniae | 4 |
| **Bacteria identified by cytobacteriological examination of sputum, blood culture, urine antigen test** |  |
| Streptococcus pneumoniae | 8^a,d^ |
| Haemophilus influenzae | 1 |
| Enterobacteriaceae | 2^e^ |
| Legionella pneumonia | 1 |
| Intracellular bacteria | 2^b^ |
| Miscellaneous | 1 |
| **Negative multiplex PCR and negative bacterial sample** | 39 |
| **Negative multiplex PCR and no bacterial sample performed** | 15 |

*Note: ^a^one patient with a co-infection Streptococcus pneumoniae + Influenza A virus, ^b^one patient with a co-infection Intracellular bacteria + Influenza A virus, ^c^one patient with a co-infection Coronavirus + Metapneumovirus, ^d^one patient with a co-infection Streptococcus pneumoniae + Rhinovirus, ^e^one patient with a co-infection Enterobacterieaceae + Rhinovirus,*
