# Supplementary material for: Diagnostic accuracy of C-reactive protein and procalcitonin in suspected community-acquired pneumonia adults visiting emergency department and having a systematic thoracic CT scan
Source: Crit Care. 2015 Oct 16;19:366. doi: 10.1186/s13054-015-1083-6 (PMC4608327; doi:10.1186/s13054-015-1083-6)

Additional file 3

Supplementary Figure: C-reactive protein (CRP) (upper panel) and procalcitonin (PCT) (lower panel) boxplot for patients with excluded CAP according to each category of alternative diagnosis. PCT values greater than 5 µg/L are not shown.

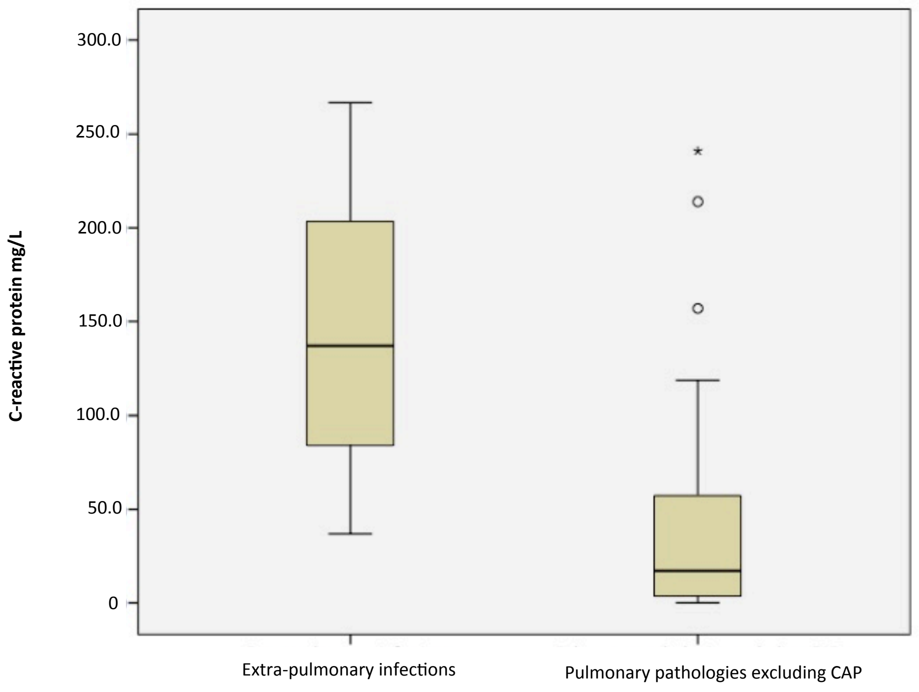

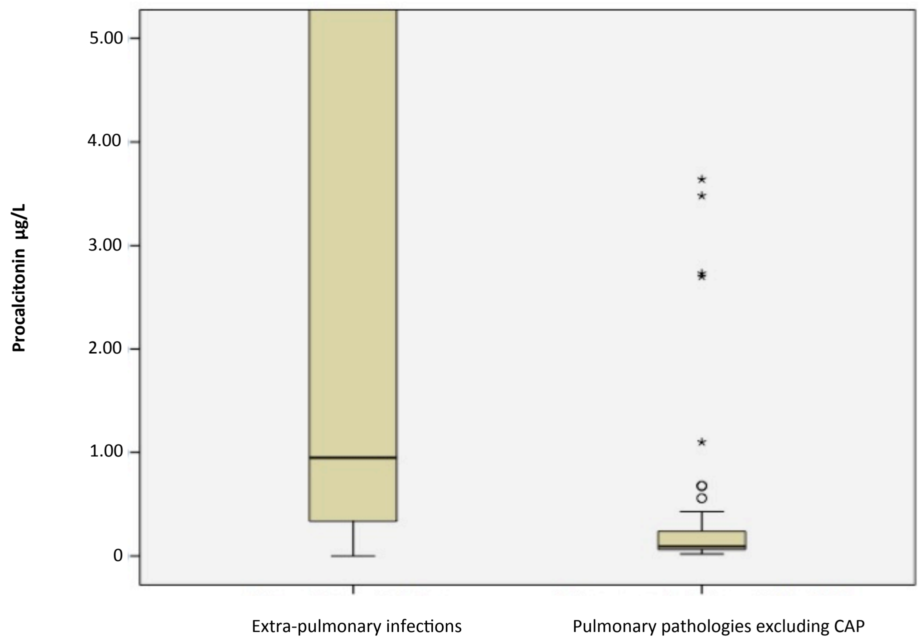

Supplement: Additional file 3: — C-reactive protein and procalcitonin boxplot for patients with excluded CAP according to each category of alternative diagnosis. (PDF 174 kb) [file 13054_2015_1083_MOESM3_ESM.pdf]
