# Supplementary material for: Diagnostic accuracy of C-reactive protein and procalcitonin in suspected community-acquired pneumonia adults visiting emergency department and having a systematic thoracic CT scan
Source: Crit Care. 2015 Oct 16;19:366. doi: 10.1186/s13054-015-1083-6 (PMC4608327; doi:10.1186/s13054-015-1083-6)

Additional file 4

Supplementary Figure: C-reactive protein (upper panel) and procalcitonin (lower panel) ROC curves predicting definite community-acquired pneumonia diagnosis (definite community acquired pneumonia versus excluded community acquired pneumonia without extra-pulmonary infections). For C-reactive protein: AUC = 0.851. CI95% = 0.790 to 0.913. Youden's Index = 0.596 for an optimal cut-off CRP point at 45.95mg/L.
For procalcitonin: AUC = 0.718. CI95% = 0.636 to 0.799. Youden's Index = 0.358 for an optimal PCT cut-off point at 0.127 μg/L.

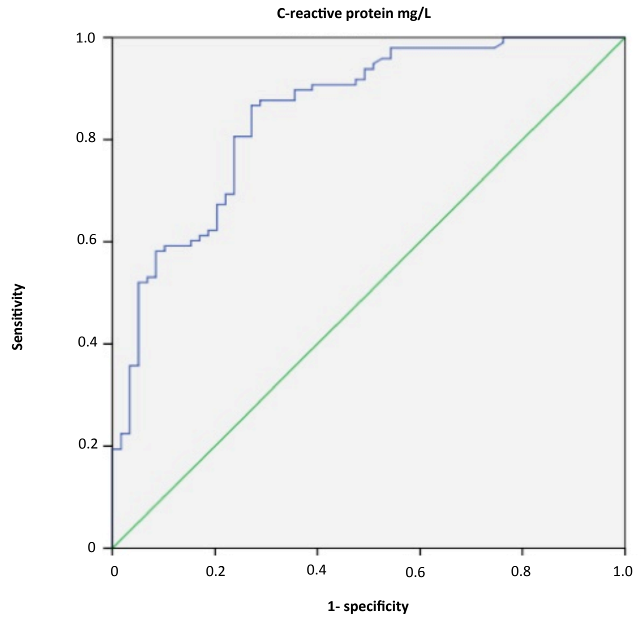

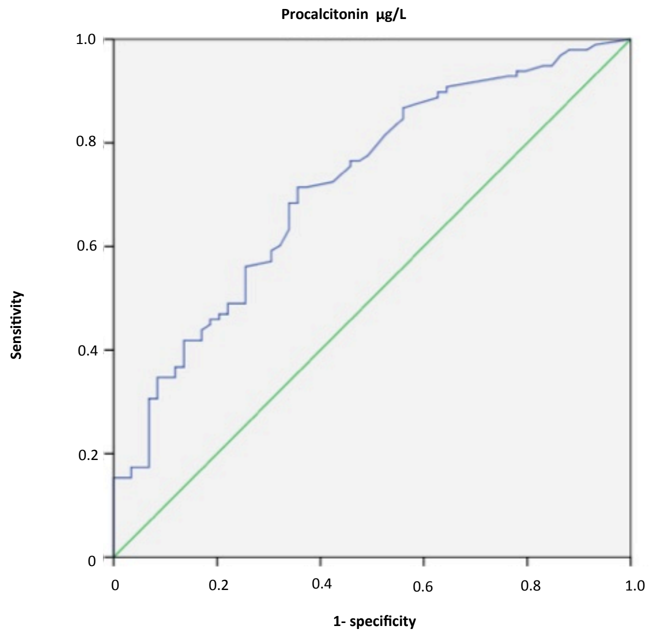

Supplement: Additional file 4: — C-reactive protein and procalcitonin ROC curves predicting definite community-acquired pneumonia diagnosis (definite community acquired pneumonia versus excluded community acquired pneumonia without extra-pulmonary infections). (PDF 176 kb) [file 13054_2015_1083_MOESM4_ESM.pdf]
